# Effect of a prescription digital therapeutic for chronic insomnia on post-treatment insomnia severity, depression, and anxiety symptoms: results from the real-world DREAM study

**DOI:** 10.3389/fpsyt.2024.1450615

**Published:** 2024-09-10

**Authors:** Frances P. Thorndike, Charles M. Morin, Joseph Ojile, Samantha Edington, Robert Gerwien, Jason C. Ong, Emerson M. Wickwire, Lee M. Ritterband, Heidi Riney

**Affiliations:** ^1^ Nox Health, Alpharetta, GA, United States; ^2^ School of Psychology and CERVO/BRAIN Research Center, Laval University, Quebec, QC, Canada; ^3^ Clayton Sleep Institute, St. Louis, MO, United States; ^4^ Statistical Consulting, Newington, CT, United States; ^5^ Division of Pulmonary, Critical Care, and Sleep Medicine, Department of Medicine, University of Maryland School of Medicine, Baltimore, MD, United States; ^6^ Department of Psychiatry, University of Maryland School of Medicine, Baltimore, MD, United States; ^7^ Center for Behavioral Health & Technology, Department of Psychiatry and Neurobehavioral Sciences, University of Virginia School of Medicine, Charlottesville, VA, United States

**Keywords:** insomnia, sleep, prescription digital therapeutic, CBT-I, real-world data, anxiety, depression

## Abstract

**Introduction:**

Chronic insomnia is a substantial public health burden that often presents with co-occurring depression and anxiety. Randomized clinical trials and preliminary real-world evidence have shown that digitally delivered cognitive-behavioral therapy for insomnia (dCBT-I) is associated with improvements in insomnia, but real-world evidence is needed to determine the true impact of digital CBT-I. This pragmatic study aimed to evaluate the benefits of treating chronic insomnia with a tailored prescription digital therapeutic in a real-world population.

**Methods:**

This prospective, single-arm clinical study involved adults aged 22-75 with chronic insomnia living in the US who had access to a mobile device. Participants accessed the FDA-cleared prescription digital therapeutic (PDT; Somryst^®^) over a 9-week intervention period. The PDT delivers cognitive-behavioral therapy for insomnia via six interactive treatment cores and daily sleep diaries used for tailoring treatment. Participants completed validated patient-reported instruments at baseline, before completing treatment cores, immediately post-intervention, and at 6-month and 1-year follow-ups. The Insomnia Severity Index [ISI], the 8-item Patient Health Questionnaire [PHQ-8], and the Generalized Anxiety Disorder-7 scale [GAD-7] were used to determine the effect of the PDT on insomnia, depression, and anxiety.

**Results:**

After screening, 1565 adults accessed the PDT. 58% of those who began the program completed Core 4, established as exposure to all mechanisms of action in the digital therapeutic. For those who completed assessments for all 6 cores (48.4%), the ISI was lowered from 18.8 to a mean of 9.9 (P <.001). These scores continued to be lower than baseline at immediate post (11.0), 6-month (11.6), and 1-year follow-ups (12.2) (P <.001). The results of the PHQ-8 and GAD-7 also show significant decreases at all measured timepoints from baseline (P <.001). Of the patients that began the program, 908 (58.0%) were considered adherent and 733 (46.8%) completed all 6 cores.

**Conclusion:**

Data from the DREAM study contributes to the growing body of clinical evidence of how patients are utilizing a PDT in the real world, outside of controlled settings, offering insights for clinicians who use these therapeutics in practice.

**Clinical trial registration:**

ClinicalTrials.gov, identifier NCT04325464.

## Introduction

Insomnia affects a large proportion of adults globally, with over a third experiencing symptoms (1, 2). In addition to nighttime sleep problems, chronic insomnia is also connected to a broad array of daytime impairments, often resulting in lower quality of life. Individuals with insomnia experience a variety of health issues, including higher rates of depression and anxiety, as well as increased likelihood of cardiovascular disease, diabetes/metabolic syndrome, hypertension, COPD, other sleep disorders, and impaired immune function (3). Further, insomnia is associated with symptoms of daytime impairment such as fatigue and cognitive impairment, leading to decreased workplace performance, higher rates of accidents, and increased risk of long-term disability — an annual economic impact estimated to have billions of dollars in hidden costs internationally (4, 5).

Cognitive Behavioral Therapy for Insomnia (CBT-I) is widely recognized and recommended as the first-line treatment option for chronic insomnia across various medical and scientific organizations including the American Academy of Sleep Medicine, American College of Physicians, European Insomnia Guideline, National Institutes of Health, and Veterans’ Affairs/Department of Defense (6–12). This evidence-based approach addresses chronic insomnia by changing the thoughts, behaviors, and habits that contribute to maintenance of sleep difficulties.

CBT-I typically involves several components (13, 14):

Sleep restriction and consolidation: limiting the time spent in bed to increase sleep efficiency before lengthening the sleep window.

Stimulus control: associating the bed and bedroom with sleep and not with wakefulness.

Cognitive restructuring: identifying and modifying beliefs that can negatively influence sleep.

In addition to the primary mechanisms of action, other treatment aspects are included. These include components like sleep hygiene education, goal setting and tracking, monitoring sleep with a sleep diary, and relapse prevention (7).

CBT-I is usually delivered over 4 to 8 sessions and can be administered individually or in groups (13, 15). Considered effective for both near-term and long-term improvement, CBT-I is beneficial not just for sleep improvement but also for reducing symptoms of other co-occurring disorders, like depression and anxiety. While generally seen as a first-line insomnia treatment due to its efficacy and more favorable risk/benefit profile as compared to pharmacotherapy (e.g., reduced risk for side effects and dependency), access to CBT-I has been limited. Barriers to care include a limited availability of trained CBT-I providers, costs/lack of insurance coverage, challenges around patient adherence and commitment to change, and language barriers (16). Digital platforms for CBT-I (dCBT-I) may be a good solution for many of these issues, as they can provide flexible and accessible options for individuals who may not have easy access to in-person therapy and multiple meta-analyses and reviews have shown comparable efficacy between electronic delivery of CBT-I and traditional modalities (17–21). These dCBT-I programs often include similar components to traditional CBT-I and are guided by evidence-based practices, although some have raised a concern that these programs will result in lower treatment adherence due to their self-directed nature, as well as lack of broad quality-control around these therapeutics, unless they are submitted for review by a third-party organization or regulatory body.

Somryst^®^, a mobile application US FDA-cleared in March 2020, is based on a dCBT-I intervention that has been extensively evaluated in over a dozen randomized trials (22, 23). The details of the Somryst PDT were previously published, outlining the evidence behind its development, details on the treatment modules, information on safety and contraindications, and data from 2 randomized controlled clinical trials submitted to FDA showing efficacy data (24).

Although the therapeutic has been FDA-cleared, continued collection of clinical data using the device paves the way for ongoing learnings that could potentially result in more informed patient care or improved outcomes. The primary objective of the Digital Real-world Evidence trial for Adults with insomnia treated via Mobile (DREAM) study is to evaluate the impact of a dCBT-I treatment on insomnia severity in a real-world setting. Secondary objectives were to examine adherence to the digital product, changes in depression symptoms, and changes in anxiety symptoms. Exploratory objectives were to identify the proportion of insomnia treatment responders and remitters.

## Methods

The research protocol, including all consent forms, was reviewed and approved by Aspire IRB on November 7, 2019 (Protocol #: PEAR-003A-101, NCT04325464). The methods for this study were previously published in full, including details on study inclusion/exclusion criteria, informed consent processes, the CBT-I treatment content, study objectives and rationale, data management, and data analysis (25). Subsequent protocol and consent revisions extended the length of time to follow participants. In brief, the study program delivered 6 treatment modules (also known as cores) to eligible patients over a 9-week period. In sequential order, these modules covered specific CBT-I content (1): overview of the CBT-I experience, (2) personalized sleep restriction and consolidation, (3) stimulus control, (4) cognitive restructuring, (5) sleep hygiene, and (6) a summary of treatment principles/relapse prevention. Of note, most of the treatment components are continued in subsequent Cores after its introduction (e.g., sleep restriction). Patients received a new core no sooner than 1 week after finishing the previous one. Patients also had to complete 5 of 7 possible sleep diaries in order to progress from Core 1 to Core 2 and to receive a new set of subsequent personalized sleep recommendations.

### Recruitment

Potential participants were recruited through a variety of sources, including patients referred by a sleep clinician or other health care provider, individuals from a study waiting list that included patients who expressed interest in a mobile version of a previous browser-based CBT-I intervention, and/or patients who conducted internet searches related to insomnia treatment.

### Assessment

Patients completed assessments, including the Insomnia Severity Index [ISI] (26, 27), before they began each core, immediately following post-intervention (63 days after initiating therapy), 6 months post-intervention (243 days after initiating therapy), and 1 year post-intervention (428 days). To be included in the study, patients had to have an ISI score of 8 or above. An 8 represents the minimum score needed on the ISI to be considered to have insomnia (8-14, subthreshold insomnia; 15-21, moderate clinical insomnia; 22-28, severe insomnia).

Additionally, patients completed the 8-item Patient Health Questionnaire [PHQ-8] (27) and Generalized Anxiety Disorder-7 scale [GAD-7] (28, 29) prior to Cores 1, 3, and 5, as well as the three follow-up timepoints (immediate post, 6 months, 1 year). The PHQ-8 (excluding the suicidal ideation item) score ranges from 0-24 (0-4, no depressive symptoms; 5-9, mild; 10-14, moderate; 15-19, moderately severe; 20-24 severe). The GAD-7 uses a 4-point scale (0, 1, 2, 3) on each of its 7 questions to assign a severity score for a patient’s anxiety (0-4, no to low risk; 5-9, mild; 10-14, moderate; 15+, severe).

### Analysis

Scores were evaluated with a mixed effect model for repeated measures (MMRM) with visit as a fixed effect and subject as a random effect. The significance of each timepoint was compared to baseline with a Dunnett test. Meaningful treatment response was assigned to any participants who reduced their ISI score by more than 7 points from baseline, and insomnia remission was defined as achieving an ISI score less than 8, indicating no clinical insomnia (25, 26).

The number of people who completed the first treatment core out of those provided with access to the dCBT-I is an important metric to gauge commitment to understanding the dCBT-I process; those patients have been termed “Core 1 completers.” Adherence was assessed by a patient completing each core; total percentage adherence compared the number of patients completing to the total that initiated treatment. An important adherence threshold is completing Core 4, when the last of the primary mechanisms of action are delivered. Completion of all elements of treatment is represented by completing Core 6. Effect sizes, using Cohen’s d, were calculated for ISI scores at immediate post intervention, and at the 6-month and 1-year follow-ups.

## Results

### Demographics of sample and baseline characteristics

Out of 1668 patients that entered the screening process, 1565 passed the inclusion criteria, began treatment with Core 1, and thus were entered into the study (**Table 1**). Patients represented all 50 US states and Washington, DC, with a mean age of 46 (standard deviation of 13.28) and were predominantly female (76.6%). Just over half of the sample (55.3%) had a baseline ISI score considered moderate (a score of 15-21); 28.3% had a severe baseline ISI and 16.3% had a subthreshold ISI. There were two individuals who qualified for the study based on their Insomnia Severity Index scores at baseline being >8 and are thus included. By the time of accessing and completing the ISI in Core 1, they reported a score that was lower than 8 and are thus marked “no insomnia” here.

**Table 1:** Demographics of sample after screening.

	N = 1565
Age, mean (SD)	46 (13.28)
Gender, n (%)	
Female Male Unknown/missing	1169 (74.7%) 345 (22.0%) 51 (3.3%)
Baseline ISI score	
No insomnia Subthreshold (ISI 8-14) Moderate (ISI 15-21) Severe (ISI 22-28)	2 (0.13%) 255 (16.3%) 865 (55.3%) 443 (28.3%)

### ISI score after core completions and follow-up

**Figure 1:**
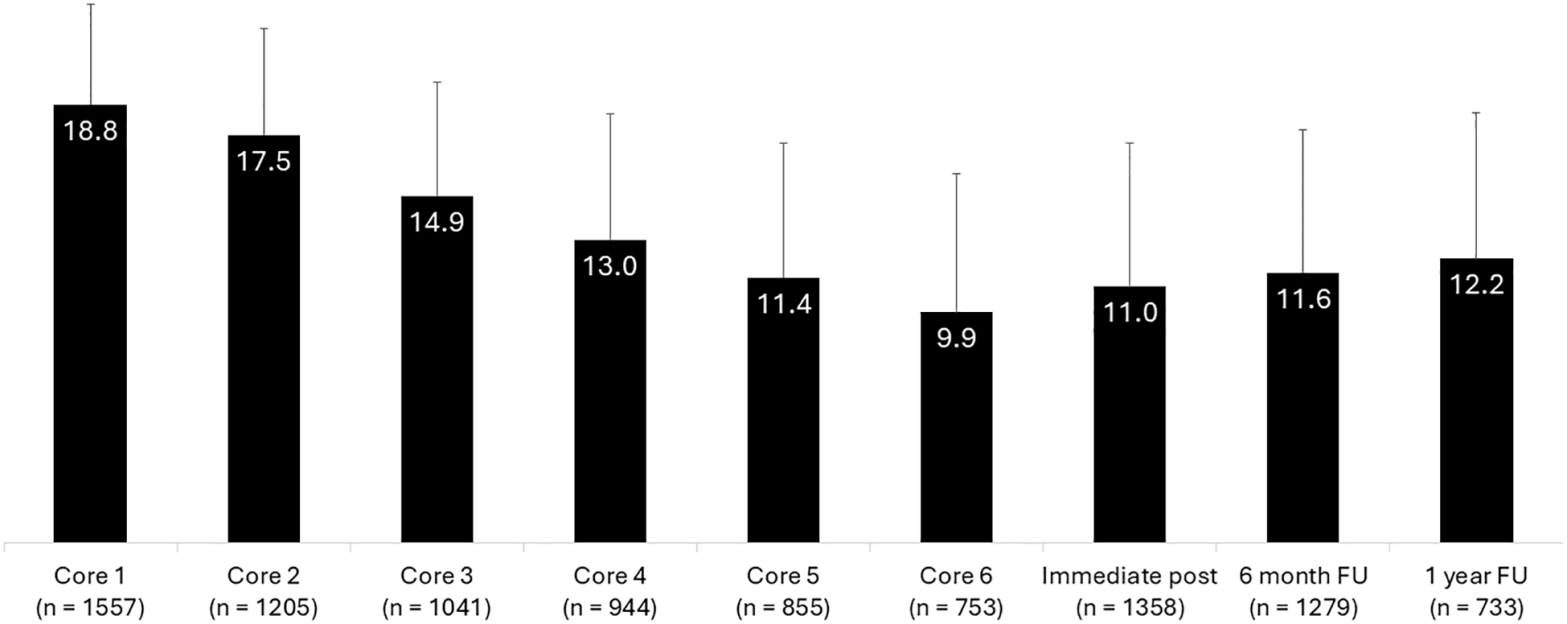
Insomnia severity index score data of participants across each treatment core and key follow-up intervals.

Prior to engaging in Core 1 of the intervention, the patients reported a mean ISI score of 18.8, indicative of moderately severe insomnia (**Figure 1**). Prior to Core 5 engagement, the timepoint of Treatment Adherence, the ISI score had lowered to a mean of 11.4. Prior to Core 6, the ISI was at its overall lowest, a mean of 9.9. From here, all participants were prompted to complete the measures regardless of amount of treatment completed. The respondents’ mean ISI was 11.0 at the immediate post and remained consistent over the 6-month (11.6) and 1-year (12.2) follow-up assessments. There was a significant reduction in ISI scores from baseline to each of the subsequent core assessments and follow-up timepoints (P <.001). In summary, the ISI decreased by nearly 9 points from baseline to Core 6 (d = 1.7), over 7 points from baseline to 6-month follow-up (d = 1.4), and 6.6 points from baseline to 1 year follow-up (d = 1.1), with all effect sizes categorized as large in magnitude.

When analyzing specifically by gender, both men and women in the study make clinically meaningful improvements. Men start at a baseline ISI of 18.8 at Core 1, move to 9.2 at Core 6, 10.8 at immediate post, 10.6 at the 6-month follow-up, and 12.4 at the 1-year follow-up (P <.001 for each timepoint from baseline). Women start at a baseline ISI of 18.9 at Core 1, move to 10.1 at Core 6, 11.1 at immediate post; 11.8 at the 6-month follow-up, and 12.0 at the 1-year follow-up (P <.001 for each timepoint from baseline).

When separating patients into severity groups according to their baseline ISI score (mild, moderate, or severe severity), we observe that each group is also able to achieve significant improvements. More specifically, patients who started the PDT with mild insomnia (ISI 8-14) began with a mean score of 12.5 at Core 1, dropped to 7.2 at Core 6, 8.0 at immediate post, 8.5 at the 6-month follow-up, and 8.5 at the 1-year follow-up (P <.001 for each timepoint from baseline). Patients with moderate severity insomnia at baseline (ISI 15-21) began with a score of 18.0 at Core 1, dropped to 9.5 at Core 6, 10.3 at immediate post, 10.9 at the 6-month follow-up, and 11.9 at the 1-year follow-up (P <.001 for each timepoint from baseline). Patients with severe insomnia at baseline (ISI > 21) began with a score of 24.1 at Core 1, dropped to 12.3 at Core 6, 14.2 at immediate post, 14.4 at the 6-month follow-up, and 15.5 at the 1-year follow-up (P <.001 for each timepoint from baseline).

### Insomnia treatment response and remission

In addition to evaluating the magnitude of the treatment effect with effect sizes, clinical significance of the treatment’s effect was also assessed according to published criteria. The proportion of insomnia treatment responders was defined as a reduction of >7 ISI score points (20). At post-intervention (Day 63), 49.2% of DREAM participants (95% CI, 46.5%–51.9%) were considered meaningful treatment responders, whereas 46.4% (95% CI, 43.6%-49.2%) and 37.8% (95% CI, 34.3%–41.5%) were considered treatment responders at 6-month and 1-year follow-ups, respectively. Clinically meaningful response rates are higher among patients who completed all 6 treatment cores: At post-intervention (Day 63), 61.8% of treatment completers (95% CI, 58.1%–65.3%) were considered meaningful treatment responders, and 56.4% (95% CI, 52.7%–60.1%) and 48.9% (95% CI, 43.8%–54.0%) were considered treatment responders at 6-month and 1-year follow-ups, respectively.

To evaluate insomnia remission, the proportion of patients who scored <8 on the ISI at a given follow-up assessment point was calculated (20). The following remission rates were observed at the respective timepoints: 32.3% (95% CI, 29.8%–34.8%) at the immediate post measurement, 28.7% (95% CI, 26.3%-31.2%) at the 6-month follow-up and 24.7% (95%CI, 21.7%–27.9%) at the 1-year follow-up. Similar to responder raters, rates of remission were also higher among those who completed all 6 treatment cores: 45.1% (95% CI, 41.5%–48.8%) at the immediate post measurement, 40.6% (95% CI, 37.0%-44.3%) at the 6-month follow-up and 33.2% (95% CI, 28.5%–38.1%) at the 1-year follow-up.

### Adherence

**Figure 2:**
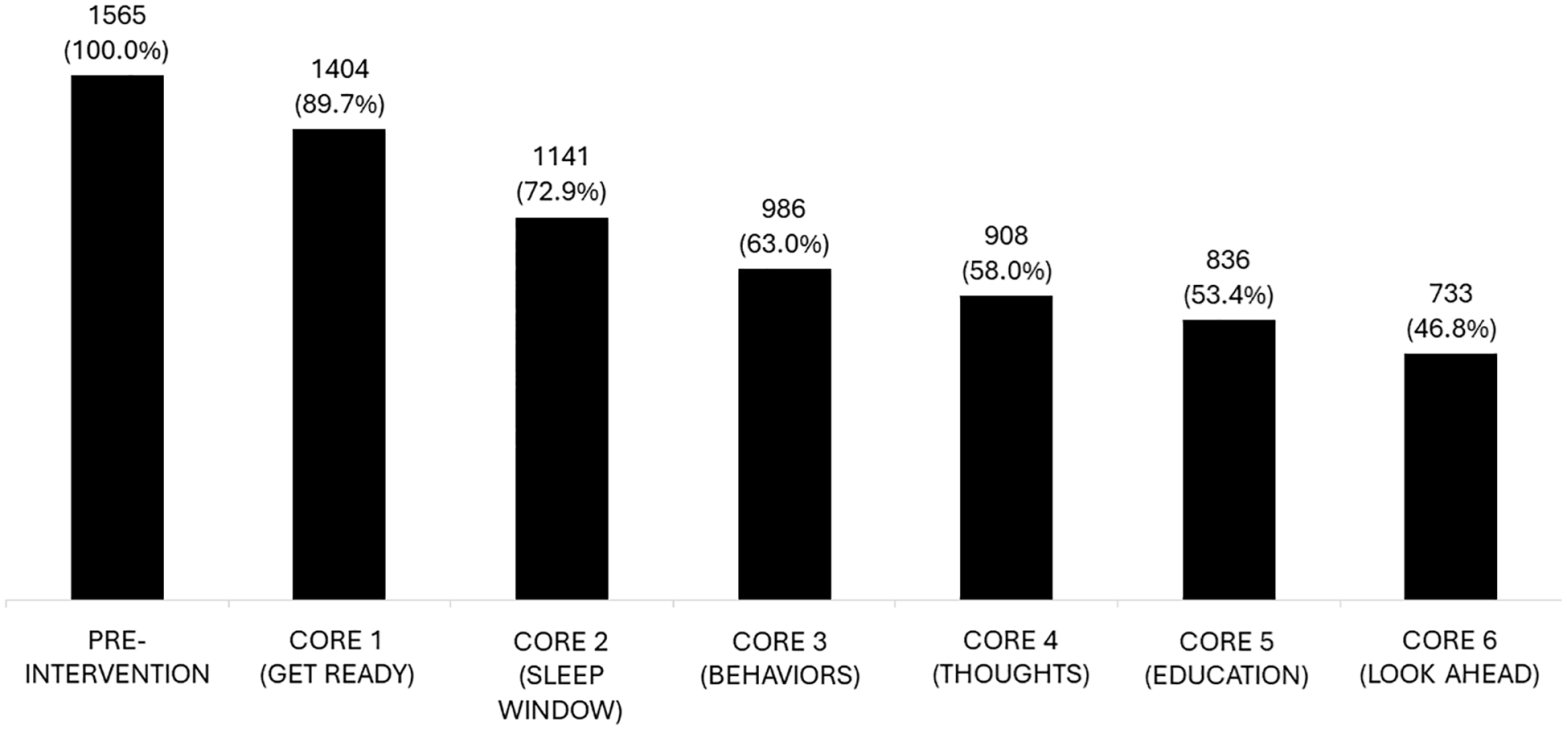
Adherence to CBT-I by treatment core modules. Of the 1565 patients that began the program, 1404 completed Core 1. Over half were considered adherent to treatment, completing at least Core 4. A total of 733 patients completed all 6 cores.

Of the 1565 patients that began the program, 1404 (89.7%) were Core 1 completers (**Figure 2**). Out of the 1565 who started the PDT, 1141 (72.9%) completed Core 2, 986 (63.0%) completed Core 3, and 908 (58.0%) completed Core 4, which indicates they received access to all mechanisms of action. Additionally, 836 (53.4%) completed Core 5, and 733 (46.8%) completed all six treatment cores.

Patients who were adherent to treatment as prescribed (completed all six treatment Cores) showed greater effect size improvement in insomnia severity than the overall sample, although both groups achieved large, meaningful improvements. The effect size of those who completed all 6 scores from baseline to the 6-month and 1-year follow-ups was large (d = 1.7 and 1.4, respectively) and the effect size of the overall sample was d = 1.4 and 1.1, respectively.

### PHQ-8 and GAD-7 scores after core completions and follow-up

The patients’ PHQ-8 scores significantly decreased from a mean of 12.3 at baseline (moderate depression) to a mean of 10.1 before Core 3, 7.8 before Core 5 (mild depression), 7.6 at the immediate post, 7.9 at the 6-month follow-up, and 8.2 at the 1-year follow-up (P <.001 comparing each timepoint to baseline).

Similarly, the patients in this study had a mean baseline GAD-7 score of 10.0, just meeting the threshold for moderate anxiety (**Table 2**). By the beginning of Core 3, the mean GAD-7 score decreased to 8.7. The GAD-7 score continued to decrease and remained at 6.9 at the beginning of Core 5, at the immediate post, and at the 6-month follow-up. At one year, the GAD-7 only increased slightly overall to a mean of 7.0 (P <.001 comparing each timepoint to baseline).

**Table 2:** Anxiety and depression score data of participants across treatment cores and key follow-up intervals.

	Core 1	Core 3	Core 5	Immediate post	6-month follow-up	1-year follow-up
PHQ8, mean (SD)	12.3 (5.24)	10.1 (5.32)	7.8 (5.40)	7.6 (5.50)	7.9 (5.64)	8.2 (5.59)
PHQ8 N	1539	1034	853	1358	1279	733
GAD-7, mean (SD)	10.0 (5.59)	8.7 (5.57)	6.9 (5.40)	6.9 (5.33)	6.9 (5.38)	7.0 (5.35)
GAD-7 N	1533	1033	852	1358	1279	733

## Discussion

Results of this national study suggest that prior findings pertaining to dCBT-I extend beyond tightly controlled clinical trials and generalize to real-world use among a diverse sample of US adults. In particular, data gathered from patients post-FDA clearance of this therapeutic yields data reasonably similar to the trials submitted to the FDA as part of seeking its clearance (23), with effect sizes in insomnia severity remaining large at all follow-up timepoints from baseline, similar to available CBT-I meta-analyses (17, 18, 21). Seyffert et al. (18) describe a meta-analysis of 4.3 points in ISI (range 2.00 to 6.65) across 15 trials, a value this trial exceeds with an observed 6.6 mean ISI decrease. As demonstrated, all groups achieved benefit from the PDT, with the severe group (who also has the most room for improvement by definition) showing the largest change in ISI score (> 7 point decrease from baseline at every time point). These findings suggest that more severe patients can meaningfully benefit from a PDT, as well as the fact that some patients may remain with residual symptoms (ISI above 8) that could potentially benefit from additional intervention, possibly booster sessions or time with a clinician.

Participants in this real-world study also showed significant reduction in symptoms of depression and anxiety. Finally, the pragmatic study demonstrated adherence comparable to the trials submitted for FDA evaluation and clearance, with the completion rate for each of the 6 treatment cores falling between the 2 trials submitted to FDA (30). In summary, both real-world and controlled settings showed large and meaningful improvements in insomnia severity, with about half of the sample completing all treatment as prescribed. Thus, these data build upon and expand prior findings pertaining to dCBT-I by adding an important real-world, pragmatic perspective and support further adoption of dCBT-I.

One difference from the RCTs observed in this real-world population was that the sample scored higher at baseline on each of the study measures. While there was a similar reduction of symptoms (with respect to ISI, PHQ-8, and GAD-7) and treatment engagement/adherence to what was seen in previous RCTs (22, 23), the real-world patient population was more symptomatic. Although these patients, on average, achieved meaningful benefit with the PDT, it is worth noting that the post-treatment average ISI score still represented mild symptoms, with fewer patients reaching full remission than observed in the RCTs. This can serve as an important reminder for clinicians that patients entering digital treatment in the real-world may be of increased severity and complexity, which is not surprising given that RCTs often have inclusion/exclusion criteria that can limit real-world generalizability. Thus, when patients access treatment with greater symptomatology it will remain important to evaluate their benefit with the digital therapeutic and determine when and how additional clinical support may be needed.

Despite positive findings, about 50% of the sample did not complete the full CBT-I program, and outcomes from the treatment completers suggest there was greater benefit for those who completed treatment as prescribed – a larger effect size on ISI reduction. Reasons for dCBT-I attrition have been previously studied, leading researchers to conclude that patients who have comorbid anxiety or depression, less severe insomnia, and a higher total sleep time prior to intervention are more likely to discontinue treatment (26, 27). However, one meta-analysis comparing format for CBT-I showed that live group CBT-I may have less program completion than dCBT-I (20). Nevertheless, efforts to continue to understand how best to support patients engaged in dCBT-I remain important and lack of these supports is noted as a key barrier to care in multiple studies (31, 32). For patients who remain symptomatic after utilizing dCBT-I, it will be important for them to remain engaged in care that continues to assess and manage treatment as warranted. Other limitations to this study include a lack of comparator group, given the design of the study to be illustrative of real-world insomnia treatment, and the longest-term follow-ups not being able to be conducted as the study ended prematurely due to loss of funding.

However, the study had multiple strengths. As a virtual trial, this study was able to happen during COVID (as it started recruiting patients in 2019) and may be more reflective of insomnia treatment in the post-COVID era. People were able to access and receive treatment without ever having to enter a “brick and mortar” clinical setting. This bodes well for reaching populations with limited access to CBT-I, alleviating some of the identified barriers to therapy initiation. Further, unlike the previous RCTs, there were few restrictions on eligibility, allowing a more generalizable reflection of the effectiveness of this digitally delivered CBT-I.

Future research should investigate further timepoints for those who have completed the entire dCBT-I program (six treatment cores) to determine if patient efforts in completing the core modules lead to a lasting effect on their ISI, PHQ-8, and GAD-7 scores. Similarly, exploring whether any baseline characteristics influence clinical outcomes can help determine who may or may not need more support to achieve optimal benefit. It will be important to continue to investigate which supplementary interventions could be added to further enhance adherence and outcomes in the dCBT-I model. Using these data as a baseline, future efforts can determine whether changes in mechanisms of follow-up and feedback to patients improve adherence across core completions, potentially leading to greater improvements in chronic insomnia. Future research will provide data to help establish standards and definitions for adherence to digital therapeutics, given the complexities, nuances, and opportunities that PDTs can provide in connecting therapeutic use with clinical outcomes.

## Data Availability

The datasets presented in this article are not readily available because of the informed consent and confidentiality restrictions. Requests to access the datasets should be directed to fthorndike@noxhealth.com.
